# Sex-Related Effects on Cardiac Development and Disease

**DOI:** 10.3390/jcdd9030090

**Published:** 2022-03-19

**Authors:** Georgios Siokatas, Ioanna Papatheodorou, Angeliki Daiou, Antigone Lazou, Konstantinos E. Hatzistergos, Georgios Kararigas

**Affiliations:** 1 School of Biology, Faculty of Sciences, Aristotle University of Thessaloniki, 54124 Thessaloniki, Greece; georsiok@bio.auth.gr (G.S.); pkioanna@bio.auth.gr (I.P.); angeldaiou@bio.auth.gr (A.D.); lazou@bio.auth.gr (A.L.); 2 Department of Cell Biology, Miller School of Medicine, University of Miami, Miami, Fl 33136, USA; 3 Interdisciplinary Stem Cell Institute, Miller School of Medicine, University of Miami, Miami, Fl 33136, USA; 4 Department of Physiology, Faculty of Medicine, University of Iceland, 101 Reykjavik, Iceland

**Keywords:** 17β-estradiol, biological sex, development, injury, regeneration, repair, sex chromosome disorders, sex determination

## Abstract

Cardiovascular diseases (CVD) are the leading cause of morbidity and mortality. Interestingly, male and female patients with CVD exhibit distinct epidemiological and pathophysiological characteristics, implying a potentially important role for primary and secondary sex determination factors in heart development, aging, disease and therapeutic responses. Here, we provide a concise review of the field and discuss current gaps in knowledge as a step towards elucidating the “sex determination–heart axis”. We specifically focus on cardiovascular manifestations of abnormal sex determination in humans, such as in Turner and Klinefelter syndromes, as well as on the differences in cardiac regenerative potential between species with plastic and non-plastic sexual phenotypes. Sex-biased cardiac repair mechanisms are also discussed with a focus on the role of the steroid hormone 17β-estradiol. Understanding the “sex determination–heart axis” may offer new therapeutic possibilities for enhanced cardiac regeneration and/or repair post-injury.

## 1. Introduction

Cardiovascular diseases (CVD) are a leading cause of morbidity and mortality [1,2]. The development, severity, progression and outcome of CVD, as well as the response to pharmacological therapies, differ between males and females [3,4,5,6,7,8]. These differences have been largely attributed to genetic and epigenetic mechanisms and to sex hormones and their receptors [3,9,10,11]. In particular, the steroid 17β-estradiol (E2) and its receptors (ER) are thought to play major roles [12,13,14,15,16,17,18].

Heart diseases are usually accompanied by significant cardiomyocyte loss, leading to acute or progressive cardiac dysfunction and ultimately to heart failure (HF). Notably, human cardiomyocytes lose their mitotic potential after birth. Thus, cardiac repair mechanisms in humans do not involve cardiomyocyte regeneration, but rather inflammatory responses and the formation of collagen-based scar tissue, both of which eventually reduce the capacity of the heart to pump blood to the body [19]. As a result, the lack of regenerative capacity of the adult human heart is considered the greatest barrier to tackling heart diseases, thereby creating an urgent need for novel regenerative strategies [20].

Animal models of cardiac regeneration provide evidence linking the capacity for cardiac regeneration with sexual maturation and reproduction. In this context, animals with plastic sexual phenotypes demonstrate full cardiac regenerative capacity [21,22]. These species are either hermaphrodites or are capable of switching phenotypic sex in response to environmental and genetic factors, such as temperature and steroid hormones [23,24]. On the other hand, in gonochoristic species with non-plastic sexual phenotypes, such as humans, sex determination and cardiomyogenesis occur exclusively during embryonic development [20]. In these species, cardiac regenerative capacity is extinguished soon after birth [25]. Intriguingly, some gonochoristic species, such as zebrafish, which are born as hermaphrodites before undergoing irreversible sex determination in their early larval life [26], retain lifelong cardiac regenerative capacity. Therefore, understanding the mechanisms that link sex determination and reproduction to cardiac regenerative capacity is of paramount importance to the field of cardiac regenerative medicine.

In the present article, we summarize current knowledge on how sex chromosome abnormalities affect cardiac development and function in a sex-dependent manner. We then discuss cardiac repair and regeneration mechanisms of organisms with cardiac regenerative properties. Lastly, sex-biased cardiac repair mechanisms in the absence of cardiac regeneration are reviewed with a focus on the role of E2.

## 2. Overview of Sex Differences in CVD

The effects of biological sex on CVD have been extensively reviewed elsewhere [10,27,28] and only a brief overview is provided here. The frequency of several cardiac disorders often differs between men and women. For instance, occlusive coronary artery disease is more common in men; however, women more frequently develop spontaneous coronary artery dissection, microvascular dysfunction, and non-obstructive coronary arteries [10,29,30]. A greater mortality rate has been reported in women following myocardial infarction; on the other hand, sudden death caused by arrhythmia is more common in men [31,32,33]. In pressure overload, there is a higher proportion of men with increased left ventricular mass and end-diastolic diameter and with decreased left ventricular relative wall thickness and function [34,35,36,37,38,39]. Notably, there is greater activation of inflammatory responses in male patients [40,41], and this response is thought to underlie male-biased cardiovascular complications in the coronavirus disease 2019 [42,43]. There are also differences in the type of HF that men and women develop. In particular, women tend to develop HF with preserved ejection fraction [44,45]; this tendency may be underlain by sex-specific remodeling of the myocardial extracellular matrix [46], and the decline in E2 at menopause might contribute to its pathogenesis [47,48]. Importantly, significant sex differences in the outcome of a variety of CVD have been reported [49,50,51].

## 3. Cardiovascular Manifestations in Sex Chromosome Disorders

A potentially informative approach towards unraveling the “sex determination–heart axis” in humans is to investigate the cardiovascular characteristics of individuals with sex-determination-related genetic abnormalities. Sex chromosome disorders are caused by abnormalities in the number or structure of sex chromosomes, i.e. X and Y. Examples include monosomy X, which leads to Turner syndrome (TS), as well as structural abnormalities of the chromosome, such as isochromosome of the long arm of the X chromosome (isochromosome Xq), which also leads to TS. Sex chromosome syndromes are associated with a range of developmental and postnatal manifestations. Here, we focus on cardiovascular abnormalities that are present in patients with Turner and Klinefelter syndromes and on the implications of the sex chromosome aneuploidy in these disorders.

### 3.1. Turner Syndrome

Originally described by Seresevskij, Ullrich and Turner, TS is a rare condition associated with partial or complete loss of one X chromosome [52,53]. Since they lack the sex-determining Y chromosome, individuals with TS develop as females and present significant fetal and neonatal death rates [54]. Females with TS who survive to birth present a clinical phenotype that may vary between younger and older individuals, with characteristics, such as short stature, infertility, hypergonadotropic hypogonadism, metabolic disorders, and increased risks of autoimmune diseases and CVD [53].

**Figure 1:**
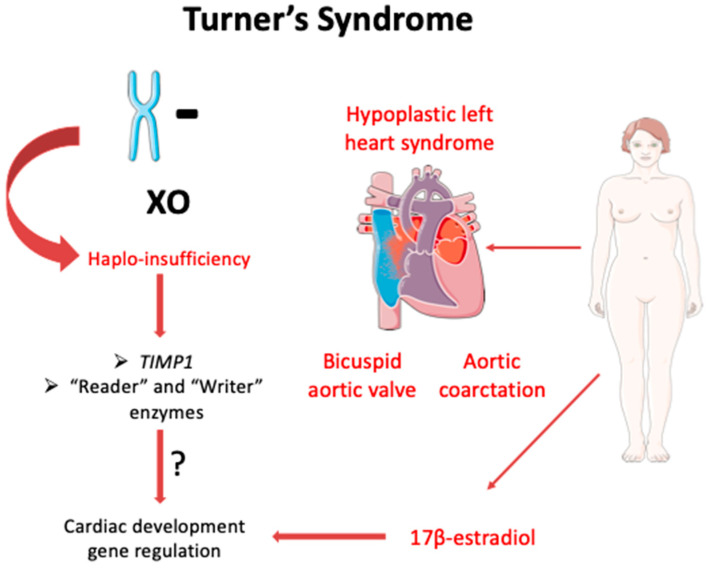
Complete or partial loss of one X chromosome in TS is associated with cardiac abnormalities. These dysfunctions vary, with hypoplastic left heart syndrome being the most common among patients with TS. Multiple factors related to the missing X chromosome have been associated with the TS phenotype. Haplo-insufficiency of *TIMP1,* “reader” and “writer” enzymes, and abnormalities in 17β-estradiol production are only part of a general genetic and hormonal instability observed in patients with TS. Figures were produced using Servier medical art.

In fact, TS is associated with a high rate of cardiovascular morbidity. Hypoplastic left heart syndrome, which has occasionally been reported in patients with TS, might be one of the factors that lead to the embryonic and neonatal deaths of individuals with TS [55] (Figure 1). Cystic hygromas are prevalent in fetuses with TS and have been implicated in cardiovascular abnormalities, such as bicuspid aortic valve (BAV) and hypoplasia of the aortic arch and left ventricular cavity [56] (Figure 1). BAV is the most common cardiovascular abnormality associated with TS and can be a risk factor for the appearance of aortic dilation and even aortic dissection [53]. Aortic coarctation (CoA) has also been reported in a large proportion of fetuses and patients with TS [57,58].

Several studies have linked cardiovascular abnormalities to the haplo-insufficiency of one or several genes on the short arm of the X chromosome (Xp) [59,60,61]. A comparative analysis of patients with non-mosaic TS has implicated Xp in congenital heart disease (CHD) that is common in women and girls with TS [59]. Moreover, a whole-genome study of patients with TS has revealed that *TIMP3*, a gene located on Xp that codes for a tissue inhibitor of matrix metalloproteinase (TIMP), is associated with aortopathy indices when hemizygous [60,62]. In addition, hemizygosity for *TIMP1*, another TIMP-coding gene, is also associated with aortopathy. Both proteins are involved in aortic valve formation. Interestingly, it has been shown that the simultaneous presence of a single copy of *TIMP1* and *TIMP3* risk alleles raises the risk of aortopathy [61] (Figure 1). TIMP imbalance is also responsible for ECM degradation that leads to excessive TGF-β signaling and the activation of SMAD signaling, both of which have been linked to an increase in fibrosis and inflammation in the aortas of patients with TS, similar to other rare disorders [63,64]. This over-activation leads to the induction of metalloproteases and eventually to aortic dilation and dissection [60].

Epigenetic processes serve a critical function and may play a key role in the TS phenotype [65]. Multiple genes important in epigenetic regulation, such as genes encoding “reader” and “writer” enzymes, are located on the X chromosome; several studies have shown differential expression in genes involved in epigenetic pathways in TS models [66,67,68]. Interestingly, an analysis of methylated regions in the leukocytes of patients with TS has revealed over 10,000 differentially methylated regions, clustered on the basis of their function in the development of congenital heart malformations and coronary heart diseases [68]. *KDM6A*, a known Y homolog and escape gene, was found to be differentially methylated between the 45,X and 46,XX karyotypes as well as significantly over-expressed in the 46,XY karyotype compared with 45,X and 45,XX [68]. Notably, KDM6A is required for appropriate cardiac cell differentiation, as it controls muscle-specific genes during myogenesis through its histone demethylase activity [69,70]. *KDM6A* differential methylation in TS could be implicated in congenital cardiovascular abnormalities associated with this syndrome [68].

Using XO mice generated as an animal model for TS, a recent study identified X-linked cardiac proteins as being differentially regulated between XO and XX mice [71]. Although these findings are important for understanding the relationship between sex chromosome proteins and cardiovascular abnormalities, species-specific differences exist in primary and secondary sex determination, gametogenesis and reproduction. In this context, XO mice exhibit a grossly normal phenotype compared with individuals with TS [72], consistent with observations that mice have a different number of X-linked genes that escape inactivation compared with humans [73,74,75]. On the other hand, human-induced pluripotent stem cells (hiPSCs) could provide a valuable model for studying the cardiac development and pathophysiology of patients with TS [76]. Notably, aneuploid hiPSC lines were used to study the early mortality and aberrant development of patients with TS [76]. Interestingly, taking into consideration the overall arrhythmias that patients with TS suffer from [77], TS hiPSCs were able to differentiate into functional cardiomyocyte-like cells, showing QT intervals comparable to control cells [76]. In addition, the expression of *CSF2RA*, a pseudoautosomal gene encoding the alpha subunit of the receptor for colony-stimulating factor 2, which is important for placenta development, has been found to be reduced in TS hiPSCs compared with the 46,XX hiPSCs [76]. This decrease in *CSF2RA* expression during early development may result in insufficient placentation in fetuses with TS, thereby leading to high mortality rates [76].

The lack of patients with non-mosaic X-del and the restricted characterization of discrete deletions in the X chromosome have hampered efforts to find more chromosomal loci that potentially contribute to the development and severity of CHD in patients with TS. Furthermore, X-Y homolog genes have not contributed to the discovery of a potential genetic mechanism for cardiac defects in patients with TS. It is possible that the answers lie in genes that escape X-inactivation, in more complex post-transcriptional mechanisms such as microRNA regulation and splicing, or in epigenetic mechanisms, which are thought to contribute to sex differences in cardiac development and malformations [65,71,78,79]. Importantly, given the gonadal insufficiency and the resulting abnormal production of sex hormones, such as E2, in patients with TS [80], it is highly likely that at least some of the TS cardiovascular abnormalities arise in response to hormonal defects. Therefore, TS hiPSC models of cardiomyogenesis could provide a useful tool, not only for functional studies [76] but also for unveiling abnormalities in the complex process of myogenesis and heart development.

### 3.2. Klinefelter Syndrome

Klinefelter syndrome (KS) was originally described in 1949 and is characterized by an extra X chromosome (genotype XXY). Because of the sex-determining Y chromosome, KS individuals are males and exhibit a range of abnormalities, including androgen deficiency, low levels of free testosterone, elevated gonadotropins and azoospermia-oligospermia [81]. In addition, multiple reports have shown that individuals with KS have a highly disturbed metabolic homeostasis and a higher mortality rate from CVD.

A cross-sectional study conducted on patients with KS, with or without testosterone treatment, and on healthy controls revealed that both insulin sensitivity deficiency and metabolic syndrome were prevalent among patients with KS. In addition, total cholesterol and low-density lipoprotein cholesterol were significantly increased; treatment with testosterone did not improve these markers [82]. Another study showed that patients with KS and metabolic syndrome typically exhibit symptoms of left ventricular dysfunction [83]. Finally, patients with KS exhibit symptoms of increased intima-media thickness, decreased peak oxygen intake and chronotropic incompetence. Again, these symptoms do not appear to be improved in patients with KS receiving testosterone therapy [84].

Although several studies have shown that the metabolic and cardiac abnormalities that appear in patients with KS are correlated with testosterone levels [82,83,85], testosterone treatment does not seem to improve the clinical phenotype [82,84]. In contrast to patients with KS receiving testosterone treatment, men with secondary hypogonadism, in the absence of KS, see considerable improvements in their cardiovascular indicators when they receive the same treatment [84]. Consequently, hormone-independent mechanisms may also contribute to the cardiovascular abnormalities of patients with KS [84]. Elucidating the role of genes for which expression and methylation differ between normal and KS hearts, or of genes that escape X inactivation, could shed light on the chromosomal mechanisms in KS. To this end, KS hiPSC-derived cardiomyocytes may be a useful tool for unraveling the underlying causes of cardiovascular abnormalities in KS [76].

## 4. Cardiac Regeneration and Repair: Effects of Biological Sex

**Figure 2:**
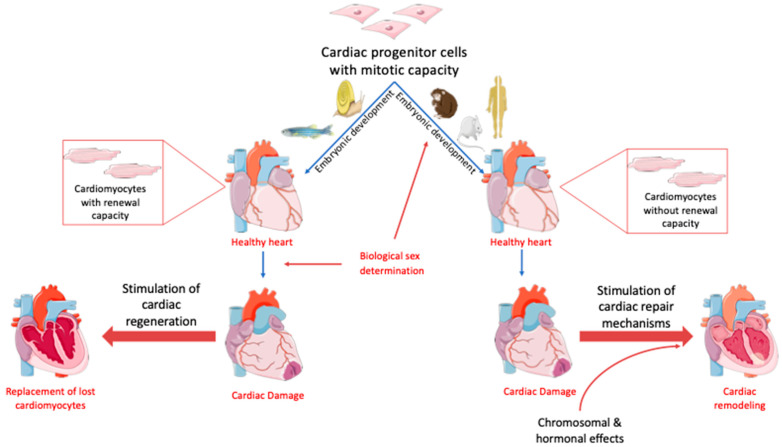
Sex determination–heart axis: sex determination is associated with cardiac regenerative capacity. Organisms with fluid sex determination, such as amphibians, zebrafish, newts and axolotls, retain cardiac regenerative capacity post-injury. In contrast, in mammalian organisms, where sex determination is permanent and takes place during development, cardiomyocyte renewal capacity is lost shortly after birth. Figures were produced using Servier medical art.

Several fish and amphibians, such as zebrafish, newts and axolotls, can completely regenerate their heart post-injury [20] (Figure 2). Zebrafish (Danio rerio) can completely regenerate their ventricle within two months after surgical amputation, and this property is retained throughout life [86]. Interestingly, a recent study suggested that female zebrafish, due to higher levels of E2, can regenerate their heart faster than male zebrafish by harnessing the immune response to cardiac injury [21]. Likewise, two photosynthetic sea slug species (*Elysia* cf. *marginata* and *Elysia atroviridis*) were recently shown to be equipped with extreme regenerative potential. These species can regrow a full body out of their autotomized heads, including a perfectly patterned and functioning heart, within nine days post-damage [22]. In contrast, the adult mammalian four-chambered heart does not have any regenerative capacity [20] (Figure 2). Interestingly, one-day-old neonatal mice can regenerate their heart post-injury, but the regenerative window closes during the first week post-birth [87]. A similar cardiac regenerative capacity has been observed in newborn pigs [88]. The fact that cardiomyocyte renewal capacity in humans is gradually lost could explain the inability of the adult mammalian heart to regenerate post-injury [89,90].

Species with cardiac regenerative capacity, such as zebrafish, exhibit plastic sex phenotypes. For example, zebrafish are born as hermaphrodites and sex determination is initiated at 28 dpf, when the animal is juvenile [91]. Sex determination may be sex-chromosome-dependent or polygenic and is also influenced by environmental factors, such as temperature and exposure to endocrine disrupting chemicals [91,92,93]. Similarly, the sea slug, which thus far exhibits the most extreme cardiac regenerative potential, is also a hermaphrodite [22]. In contrast, primary and secondary sex determination in humans occur during embryonic development in a Y-chromosome-dependent manner. Interestingly, a recent study suggested that cardiac differences between males and females first appear at the embryo stage, thus following a sex-chromosome-dependent mechanism [71]. This early sex determination may account for the inability of humans to generate new cardiomyocytes (Figure 2).

As the human heart is unable to regenerate, research efforts have focused on the elucidation of mechanisms underlying cardiac repair. To date, there is a plethora of data indicating that biological sex affects the capacity for cardiac repair, as reviewed elsewhere [10,27,94]. Here, we provide a brief overview of recent findings. In this context, a recent study reported sex differences in wound revascularization in a graded-ischemia mouse model [95]. It was noted that higher expression of *Fgf2* and *Notch1* in female mice promoted endothelial proliferation, migration and differentiation, resulting in more efficient local angiogenesis and revascularization. Among other molecules, FGF has been suggested as having a potential role in regulating cardiomyocyte proliferation and thus in repairing the injured heart [96]. Female mice have higher survival rates after myocardial infarction than male mice, with the improved cardiac function and recovery found to be associated with higher levels of arachidonic-acid-derived cypoxins and higher expression of cyclooxygenase [94]. Another factor that is correlated with myocardial ischemia is tumor necrosis factor alpha (TNFα) along with its receptor TNFR1 [97,98]. In particular, TNFα was found to promote cell migration only in cardiac progenitor cells (CPCs) from female patients [97], while female mouse hearts appear to be resistant to TNFR1 signaling during ischemia [98].

## 5. Role of E2 in Cardiac Injury and Repair

E2 is a member of the estrogen family and governs the female reproductive cycle and the development of secondary sex characteristics [99]. E2 is primarily produced in the ovaries. However, there are also extra-ovarian sources of E2. These include the adipose, breast and adrenal tissues; the bones; the heart; the brain; and the skin, where aromatase can be produced [100]. In addition, the testes and the prostate are production sites of E2 through the local conversion of androgenic precursors by the aromatase enzyme. E2 exerts its effects by binding and activating ERα and ERβ, as well as the G-coupled estrogen receptor (GEPR), in genomic and non-genomic actions [99,101,102].

A plethora of studies have reported that the E2/ER axis exerts vast effects on the cardiovascular system [103,104,105,106,107,108,109,110,111,112,113,114,115,116,117] and that these effects can be sex-dependent [14,118,119,120,121,122]. In this context, several pre-clinical data support the notion that E2 facilitates endogenous cardiac repair processes in animal models of ischemia/reperfusion (I/R) and myocardial infarction (MI) [101,123]. Importantly, ovariectomized (OVX) animals demonstrate decreased post-ischemic functional recovery and increased mitochondrial and cardiomyocyte damage, whereas E2 treatment reverses these effects [13,124,125]. The underlying molecular mechanisms are both genomic and non-genomic [101,102,123] and include modulation of ion channel activity [104,124,126], antioxidant effects [101,127,128], anti-inflammatory or anti-apoptotic effects [129,130,131], and effects on endothelial cells [132,133]. Importantly, a significant number of E2-mediated effects involve alterations in PI3K signaling [134,135,136]. The E2/ER axis has been shown to influence cardiac responses to damage by mechanisms that are also dependent on microRNA actions [16,137], ultimately leading to increased functional recovery and repair. Nevertheless, there have also been some conflicting data, with a few reports indicating that E2 treatment could also have adverse effects on cardiac repair. In particular, E2 was associated with increased cardiac damage and mortality in the acute phase post-MI; however, it was associated with improved cardiac repair and increased survival in the long term [133]. On the other hand, others reported that even though E2 treatment was accompanied by reduced infarct size in the acute phase post-MI, it failed to prevent cardiac damage [138] or was even associated with increased damage and mortality in the long term [139]. Furthermore, it was shown that the administration of E2 led to opposite effects in two different strains of mice [110]. Species and genetic model differences, as well as different methods of hormone administration, offer some possible explanations for these discrepancies. In addition, the existence of a large variety of endpoints on which cardiac repair is assessed makes comparisons more difficult.

The discovery of the presence of ER in many types of stem cells [140] offered another approach to utilizing E2 for myocardial repair. E2 treatment of mesenchymal stem cells (MSCs) [141,142], bone-marrow-derived endothelial progenitor cells (EPCs) [143] or cardiac stem cells (CSCs) [144] enhanced the functional potential of these cells for myocardial repair and resulted in improved functional recovery during ex vivo I/R. These effects could be partially attributed to the induction of migration and homing factors [145,146] as well as to the proliferative capacity of CSCs [147]. Surprisingly, the effects of E2 on hiPSC-derived cardiomyocytes are currently largely unexplored, despite the fact that female hiPSCs differ from the male version in their sex steroids and autosomal gene expression [148]. Although the clinical efficacy of stem-cell-based therapies for myocardial damage has been questioned [149], hiPSCs provide a novel and valuable tool for achieving myocardial repair [20]. The elucidation of E2 effects on hiPSC function and differentiation could shed some light on the regulatory role of E2 in the myocardium and provide new opportunities for utilizing stem-cell-based therapies for cardiac repair.

## 6. Conclusions

Biological sex is implicated in various pathophysiological responses of different organs, including the heart, not only in humans but also in other species. Sex chromosome disorders, such as Turner and Klinefelter syndromes, are characterized by a wide variety of cardiovascular manifestations, providing an excellent example of the role of sex chromosomes in the development and physiology of the heart. Given the high regenerative capacity of asexually reproducing animals and hermaphrodites, it may be inferred that sex determination processes affect mechanisms of heart development and regeneration. Further research is necessary to better understand the role of primary and secondary sex determination mechanisms in cardiovascular pathophysiology. These, in turn, could contribute to the development of novel regenerative therapies.

## Data Availability

Not applicable.
